# Supplementary material for: Airway epithelial interferon response to SARS-CoV-2 is inferior to rhinovirus and heterologous rhinovirus infection suppresses SARS-CoV-2 replication
Source: Sci Rep. 2022 Apr 28;12:6972. doi: 10.1038/s41598-022-10763-2 (PMC9048621; doi:10.1038/s41598-022-10763-2)
Supplement: Supplementary file 1 — Supplementary Figure 1. [file 41598_2022_10763_MOESM1_ESM.pdf]

Supplementary Information

Airway epithelial interferon response to SARS-CoV-2 is inferior to rhinovirus and heterologous rhinovirus infection suppresses SARS-CoV-2 replication

Elizabeth R. Vanderwall[1], Kaitlyn A. Barrow[1], Lucille M. Rich[1], David F. Read[2], Cole Trapnell[2], Oghenemega Okoloko[1], Steven F. Ziegler[3], Teal S. Hallstrand[4], Maria P. White[1], Jason S. Debley[1,5]

[1] Center for Immunity and Immunotherapies. Seattle Children's Research Institute, Seattle, WA., USA.

[2] Department of Genome Sciences. University of Washington, Seattle, WA., USA.

[3] Center for Fundamental Immunology, Benaroya Research Institute at Virginia Mason, Seattle, Washington; Department of Immunology, University of Washington School of Medicine, Seattle, Washington.

[4] Division of Pulmonary, Critical Care, and Sleep Medicine and the Center for Lung Biology, University of Washington, Seattle, WA., USA.

[5] Department of Pediatrics, Division of Pulmonary and Sleep Medicine, Seattle Children's Hospital, University of Washington, Seattle, WA., USA.

Supplement Figure 1. Panels A and B: Organotypic AEC cultures from 3 pediatric donors were infected with SARS-CoV-2 WA-01, SARS-CoV-2 Delta, or SARS-CoV-2 Omicron (red boxplots) at MOIs of 0.5 or 0.1 or with HRV-16 (blue box plots) at MOIs of 0.5 or 0.1. RNA was harvested from AEC cultures (in triplicate from each donor AEC line) 96 hours following infection with SARS-CoV-2 strains or HRV-16 and PCR was run to measure expression of *IFNB1* **(Panel A)** or *IFNL2* **(Panel B)** relative to the housekeeping gene *GAPDH*. **Panels C and D:** In cultures from 3 pediatric donors, AECs were infected either with SARS-CoV-2 alone (red boxplots) or with HRV-16 followed 72 hours later by infection with SARS-CoV-2, using three different MOIs (0.5, 0.1, 0.01) of HRV-16 pre-infection (blue box plots) followed by infection with SARS-CoV-2 WA-01, SARS-CoV-2 Delta, or SARS-CoV-2 Omicron at an MOI of 0.5. RNA was harvested from AEC cultures (in triplicate from each donor AEC line) 96 hours following infection with SARS-CoV-2 strains or HRV-16 and PCR was run to measure expression of *IFNB1* **(Panel C)** or *IFNL2* **(Panel D)** relative to the housekeeping gene *GAPDH*. Friedman ANOVA for experiments in panels A, B, C, and D were all $p<0.0001$. Post hoc Dunn's tests: *$p<0.05$, **$p<0.01$, ***$p<0.001$, ****$p<0.0001$.

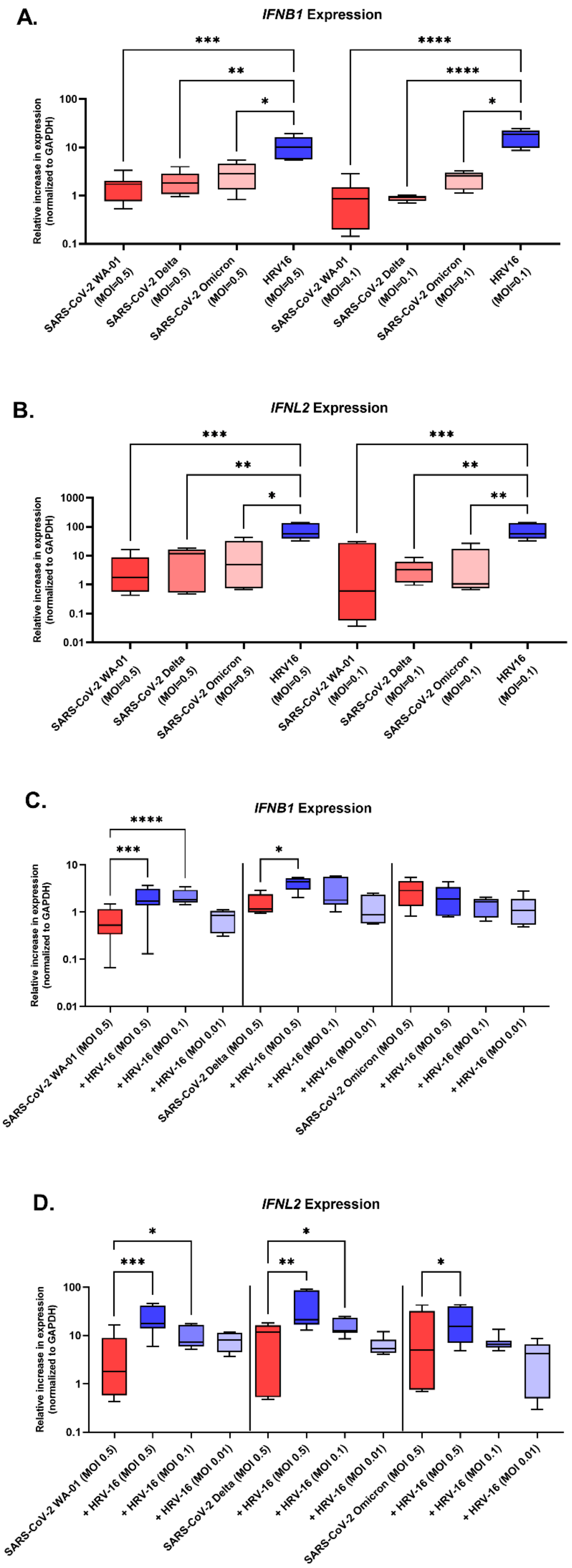
A.
IFNB1 Expression
Relative increase in expression (normalized to GAPDH)
100
10
1
0.1
***
**
*
****
****
*
SARS-CoV-2 WA-01 (MOI=0.5)
SARS-CoV-2 Delta (MOI=0.5)
SARS-CoV-2 Omicron (MOI=0.5)
HRV16 (MOI=0.5)
SARS-CoV-2 WA-01 (MOI=0.1)
SARS-CoV-2 Delta (MOI=0.1)
SARS-CoV-2 Omicron (MOI=0.1)
HRV16 (MOI=0.1)
B.
IFNL2 Expression
Relative increase in expression (normalized to GAPDH)
1000
100
10
1
0.1
0.01
***
**
*
***
**
**
SARS-CoV-2 WA-01 (MOI=0.5)
SARS-CoV-2 Delta (MOI=0.5)
SARS-CoV-2 Omicron (MOI=0.5)
HRV16 (MOI=0.5)
SARS-CoV-2 WA-01 (MOI=0.1)
SARS-CoV-2 Delta (MOI=0.1)
SARS-CoV-2 Omicron (MOI=0.1)
HRV16 (MOI=0.1)
C.
IFNB1 Expression
Relative increase in expression (normalized to GAPDH)
10
1
0.1
0.01
****
***
*
SARS-CoV-2 WA-01 (MOI 0.5)
+ HRV-16 (MOI 0.5)
+ HRV-16 (MOI 0.1)
+ HRV-16 (MOI 0.01)
SARS-CoV-2 Delta (MOI 0.5)
+ HRV-16 (MOI 0.5)
+ HRV-16 (MOI 0.1)
+ HRV-16 (MOI 0.01)
SARS-CoV-2 Omicron (MOI 0.5)
+ HRV-16 (MOI 0.5)
+ HRV-16 (MOI 0.1)
+ HRV-16 (MOI 0.01)
D.
IFNL2 Expression
Relative increase in expression (normalized to GAPDH)
100
10
1
0.1
*
***
*
**
*
SARS-CoV-2 WA-01 (MOI 0.5)
+ HRV-16 (MOI 0.5)
+ HRV-16 (MOI 0.1)
+ HRV-16 (MOI 0.01)
SARS-CoV-2 Delta (MOI 0.5)
+ HRV-16 (MOI 0.5)
+ HRV-16 (MOI 0.1)
+ HRV-16 (MOI 0.01)
SARS-CoV-2 Omicron (MOI 0.5)
+ HRV-16 (MOI 0.5)
+ HRV-16 (MOI 0.1)
+ HRV-16 (MOI 0.01)
